# CD11b Expression Level Measurement to Distinguish Asthma Phenotype in Pediatric Patients

**DOI:** 10.1055/s-0045-1802666

**Published:** 2025-04-02

**Authors:** Tien Budi Febriani, Soeroyo Mahfudz, Emi Azmi Choironi, Ninda Devita, Adika Zhulhi Arjana, Muhammad Nur Imansyah

**Affiliations:** 1 Department of Pediatric, Faculty of Medicine, Universitas Islam Indonesia, Yogyakarta, Indonesia; 2 Department of Microbiology, Faculty of Medicine, Universitas Islam Indonesia, Yogyakarta, Indonesia; 3 Department of Clinical Pathology, Faculty of Medicine, Universitas Negeri Yogyakarta, Yogyakarta, Indonesia; 4 Faculty of Medicine, Universitas Negeri Yogyakarta, Yogyakarta, Indonesia

**Keywords:** asthma phenotype, pediatric asthma, CD11b

## Abstract

**Background** Inflammation is involved in asthma pathogenesis. Based on the majority of inflammatory cells found in blood samples, asthma is divided into neutrophilic and eosinophilic phenotype. CD11b is a leukocyte surface antigen that is involved in the inflammation process. However, the immunologic response mechanism in asthma phenotypes, especially in pediatric population, is still unclear. The aim of this study was to find the difference of CD11b expression in neutrophilic and eosinophilic phenotype of asthma in pediatric patients.

**Methods** This is an analytic nonexperimental study. Data are gathered with cross-sectional method. Study subject is pediatric patients (5–17 years old) with asthma in Persaudaraaan Djamaah Hadji Indonesia and Wonosari hospitals. Blood samples for leukocyte differential count were obtained from patients who met the inclusion and exclusion criteria. Samples were analyzed with flow cytometry to measure the CD11b level. The obtained data was analyzed with independent *t* -test with Medcalc software.

**Result** A total of 40 patients were enrolled in this study. Based on blood sample analysis, 70% patients had neutrophilic asthma and 30% patients had eosinophilic asthma. There was no significant difference between patient ages mean in both groups (7.071 vs. 7.471; *p* = 0.7186). The mean of leukocyte count was significantly higher in patients with neutrophilic asthma, compared with eosinophilic asthma (8.468 vs. 4.817 × 10 ^9^ /L; *p* = 0.0008). CD11b expression in neutrophilic asthma was significantly higher than eosinophilic asthma ( *p* = 0.046). No association was found between CD11b expression levels with age, sex, and body mass index.

**Conclusion** CD11b expression level is higher in neutrophilic phenotype compared with the eosinophilic phenotype in pediatric asthma patients. Its increased expression could provide insights into the mechanisms driving airway inflammation and remodeling. This marker might be explored as a target for novel therapies aimed at modulating immune activation to reduce asthma exacerbations and improve long-term outcomes.

## Introduction

Asthma refers to a reversible narrowing of the airways. 1 This condition ranks among the most prevalent chronic illnesses globally, impacting an estimated 300 million individuals currently, with projections suggesting a potential increase to 100 million more people affected by 2025. 2 On a global scale, childhood asthma-related fatalities range from 0 to 0.7 per 100,000 individuals. Among children, asthma stands as one of the most widespread chronic ailments, featuring among the top 20 conditions contributing to disability-adjusted life years in this demographic. 3 In Indonesia, the prevalence of current wheezing in adolescents rose from 2.1% in 1996 to 5.2% in 2002 before stabilizing at 4.6% in 2016, although this figure might reflect the actual scenario or potentially stem from underdiagnosis or underreporting. A study in Yogyakarta showed both children and adolescents to have a comparable prevalence of 4.6% for current wheezing. 4

Asthma, traditionally classified as hypersensitivity type-1, is associated not only with hypersensitivity but also with inflammation and the coagulation process, as indicated by recent research findings. 5 It can now be broadly categorized into eosinophilic or neutrophilic types based on the cellular profiles in the airways or blood. Sputum analysis is preferred in asthma research to determine its type, with eosinophilic asthma characterized by increased eosinophils (over 2 or 3%) and neutrophilic asthma by heightened neutrophil levels (above 60 or 76%) in induced sputum. 6 7 However, the counts of eosinophils/neutrophils in sputum showed a positive correlation with the counts of eosinophils/neutrophils in the blood. 8

Neutrophilic asthma seems to be triggered by innate immune pathways, primarily involving type 2 inflammatory pathways where immunoglobulin E-mediated signals have a less significant role. 6 This form of airway inflammation is primarily caused by Th1 and Th17 lymphocytes. 1 Factors such as exposure to air pollutants, obesity, a high-fat diet, cigarette smoking, and respiratory infections contribute to this mechanism. 9 10

Neutrophils are implicated in causing bronchial hyperresponsiveness. Neutrophil elastase, for instance, can lead to increased mucus secretion, mucus gland hyperplasia, and impact the growth or death of airway smooth muscle cells. 11 Neutrophils can cause further negative effects on the air passages, such as the narrowing of airways through airway restructuring and a swift reduction in lung functionality. 12 Children with severe asthma who are unresponsive to treatment have high sputum neutrophil counts. 13 12 In a 12-year follow-up, children diagnosed with neutrophilic asthma needed higher doses of inhaled corticosteroids and had more unplanned respiratory visit. 14

The movement of neutrophils from circulation to inflamed areas involves controlled expression of leucocyte surface adhesion molecules. L-selectin (CD62L), crucial for the initial attachment of leucocytes to the endothelium, is quickly shed after neutrophil activation. This is followed by granulocyte migration facilitated by β2 integrins such as CD11b, primarily present in neutrophils. 11 CD11b plays a role in leukocyte adhesion and movement during inflammation, and it functions as a C3bi complement receptor involved in complement uptake. 15 16

However, children exhibiting signs of neutrophilic asthma seem to exhibit distinct inflammatory mediator profiles compared with adults experiencing the same condition. 17 In adults with neutrophilic asthma, heightened levels of sputum interleukin (IL)-17A, a proinflammatory cytokine responsible for recruiting neutrophils through T cell mediation, are linked to the severity of the illness. 18 Nonetheless, although elevated in children with severe asthma, there is not a direct association found between its levels and the severity of the disease in this younger population. 19 20 Consequently, this study aims to discern the differences in CD11b expression between neutrophilic and eosinophilic asthma phenotypes in pediatric patients.

## Material and Methods

### Study Design

This was an analytic nonexperimental study to assess the differences in CD11b expression between neutrophilic and eosinophilic pediatric asthma patients without providing treatment or therapy. The data were obtained with a cross-sectional method from November 2018 to May 2019 in Wonosari Hospital and Persaudaraaan Djamaah Hadji Indonesia Hospital, Yogyakarta, Indonesia. This study was approved by the Ethics Committee of the Faculty of Medicine, Islamic University of Indonesia with Letter No. 15/Ka.Kom.Et/70/KE/I/2018.

### Subject Recruitment

The study subjects were children 5 to 17 years old recruited from the emergency department. Subjects were diagnosed with asthma if they meet the following criteria: wheezing is found on physical examination, complaints improve with nebulization, and there is a history of previous attacks. 21 The diagnosis is made by the emergency room doctor on duty when the patient is admitted to the hospital. Subjects were excluded from the study if they refused to participate and if also diagnosed with other diseases.

Oral and written informed consents were obtained from the subjects. Informed consent was given by the emergency room doctor to the patient's guardian using an informed consent form that had been created by the researcher. Then, subjects were divided into neutrophilic and eosinophilic asthma based on the dominant type of leukocyte on routine blood tests. 7 Neutrophilic asthma is an asthma in a patient whose predominance is neutrophils on routine blood tests. Eosinophilic asthma is an asthma in a patient who is predominantly eosinophilic on routine blood tests. Routine blood results are obtained from medical record data.

The sample size for this study was calculated using the sample size formula for unpaired numerical analysis. The minimum required sample size was determined to be 18 per group. Type I error is set at 5%, type II at 10%, minimum meaningful difference at 20, and standard deviation (SD) at 20. 22 The sampling technique used consecutive sampling.

### Blood Sampling

Subject blood samples were collected in a volume of 100 µL and immediately placed into tubes containing ethylenediaminetetraacetic acid anticoagulant. The samples were then stored in a refrigerator at a temperature of 2 to 8°C to maintain their stability.

### Flow Cytometry Assay

The flow cytometry examination was performed at the Department of Clinical Pathology, Faculty of Medicine, Public Health and Nursing, Universitas Gadjah Mada, Yogyakarta, Indonesia. CD11b expression was counted with FACSCanto flow cytometer using BD CD11b PE (BD Bioscience).

A specific amount of CD11b antibody reagent was added to 100 µL of whole blood in a 12 × 75 mm tube. The mixture was carefully vortexed and incubated in the dark at room temperature (20–25°C) for 15 to 30 minutes. After incubation, 2 mL of fluorescence-activated cell sorting lysing solution was added, followed by gentle vortexing. The mixture was then incubated again in the dark at room temperature for 10 minutes. The sample was centrifuged at 500 × *g* for 5 minutes, and the supernatant was discarded. A volume of 2 to 3 mL buffer solution was added, and the sample was centrifuged again at 500 × *g* for 5 minutes. After removing the supernatant, 0.5 mL of 1% paraformaldehyde solution was added to the pellet and thoroughly mixed to ensure proper fixation. The prepared sample was then loaded into the flow cytometry instrument for analysis. Flow cytometry results for CD11b level were shown in cells/mm ^3^ . The percentage of CD11b expression shows the percentage of neutrophils that express CD11b, so the absolute value is the absolute number of neutrophils that express CD11b.

### Statistical Analysis

Variable data with a categorical scale is displayed with frequency and proportion. Variable data with a continuous scale is displayed in mean ± SD if the distribution is normal. If the data distribution is not normal, the data are displayed in median (min–max). Categorical data are analyzed using the chi square test. Data with a continuous scale is analyzed using the independent *t* -test if the data distribution is normal. If the data are not normally distributed, it is analyzed using the Mann–Whitney test. The normality test uses the Kolmogorov–Smirnov test.

The independent variable was asthma phenotype (as categorical data), and meanwhile the dependent variable was the CD11b level (as numeric data). The CD11b data from the eosinophilic and neutrophilic asthma group were compared with an independent *t* -test. Linier regression analysis was performed to assess other factors influencing the CD11b level. A *p* -value of < 0.05 was considered statistically significant. Analyses were performed with Medcalc for the Windows operating system.

## Results

A total of 40 subjects were enrolled in this study, with 70% patients in the neutrophilic asthma group and 30% patients in the eosinophilic asthma group. As shown in Table 1 , there was no significant difference in the subject's age among both groups ( *p* = 0.7186). Most of the neutrophilic asthma group subjects were males (67.86%), meanwhile the eosinophilic asthma group was mostly females (58.33%). There were no significant differences in clinical manifestations, both anamnesis and physical examination, between the two groups. A history of allergy was obtained from all the subjects.

**Table 1:** Baseline characteristics and clinical manifestation difference between subjects with neutrophilic asthma and eosinophilic asthma

	Neutrophilic asthma *N* = 28	Eosinophilic asthma *N* = 12	*p*
Age (y)	7.071 ± 2.4026	7.417 ± 3.4761	0.7186
Sex, *n* (%)			0.2312
Female	9 (32.14)	7 (58.33)	
Male	19 (67.86)	5 (41.67)	
BMI	17.19 ± 1.90	17.71 ± 2.38	0.595
Chief complaint, *n* (%)			
Duration	100.5 (32–180)	97 (30–176)	0.6159
Breathless	9 (32.14)	6 (50)	0.4760
Wheezing	16 (57.14)	5 (41.67)	0.5804
Chest pain	15 (53.57)	7 (58.33)	0.9447
Shortness of breath	18 (64.29)	5 (41.67)	0.3285
History, *n* (%)			
Allergy	28 (100)	12 (100)	
Paroxysmal cough	14 (50)	7 (58.33)	0.8901
Chronic cough	13 (46.43)	8 (66.67)	0.4070
Family history, *n* (%)			
Paternal allergy	14 (50)	4 (33.33)	0.5325
Maternal allergy	16 (57.14)	4 (33.33)	0.3006
Siblings with allergy	9 (32.14)	6 (50)	0.4760
Physical examination, *n* (%)			
Heart rate	102.929 ± 5.8495	102.917 ± 6.2879	0.9954
Respiratory rate	28.321 ± 2.2942	27.833 ± 3.0101	0.5782
Retraction	15 (53.57)	7 (58.33)	0.9447

Abbreviations: BMI, body mass index; SD, standard deviation.

Note: Data are presented as *n* (%), means (SDs), or medians (interquartile ranges). Statistical significance was evaluated by independent *t* -test for numeric variables and chi-square test for categorical variables. *p* -Value is significant if < 0.05.

Complete blood examination results showed that the mean number of leukocytes in subjects with the neutrophilic asthma phenotype was significantly higher than the eosinophilic asthma phenotype (8,468 vs. 4,817; *p* = 0.0088). Likewise, significant differences were found in the parameters of percent neutrophils and percent eosinophils ( *p* = 0.0094 and < 0.0001) ( Table 2 ).

**Table 2:** Comparison of complete blood examination results between subjects with neutrophilic asthma and eosinophilic asthma

	Neutrophilic asthma *N * = 28	Eosinophilic asthma *N* = 12	*p*
Leukocyte count (×10 ^9^ /L)	8.468 ± 4.3563	4.817 ± 2.0211	0.0088 a
Hemoglobin (g/dL)	11.9 (7.9–26.5)	11.75 (10.5–16.9)	0.8943
Hematocrit (%)	36.1 (27.2–80.6)	34.7 (30.4–52.5)	0.7454
Platelet count (×10 ^3^ /µL)	238.036 ± 139.6364	270.25 ± 65.4135	0.452
Neutrophil %	70.35 (17.5–89.2)	57.45 (29.9–79.8)	0.0094 a
Lymphocyte %	22.15 (6.4–70.5)	26.75 (3.5–50.7)	0.2814
Monocyte %	7.2 (3.8–17.2)	7.2 (3.6–13)	0.9412
Eosinophil %	0.1 (0–0.9)	7.5 (5.1–12.3)	< 0.0001 a
Basophil %	0.25 (0–1.5)	0.4 (0–1.6)	0.884

Abbreviation: SD, standard deviation.

Note: Data are presented as means (SDs) or medians (interquartile ranges). Statistical significance was evaluated by independent *t* -test.

a *p* -Value is significant if < 0.05.

**Fig. 1:**
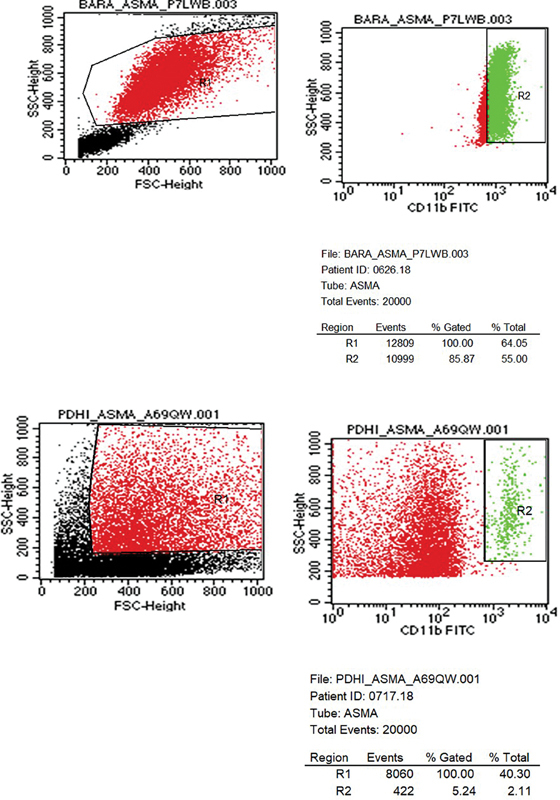
Gating strategy for CD11b expression. Gating strategy used to identify CD11b expression using flow cytometry. CD11b ^pos^ was gated from both high side scatter (SSC) and forward scatter (FSC). Previous negative control was used to ensure the correct gating of all samples.

**Fig. 2:**
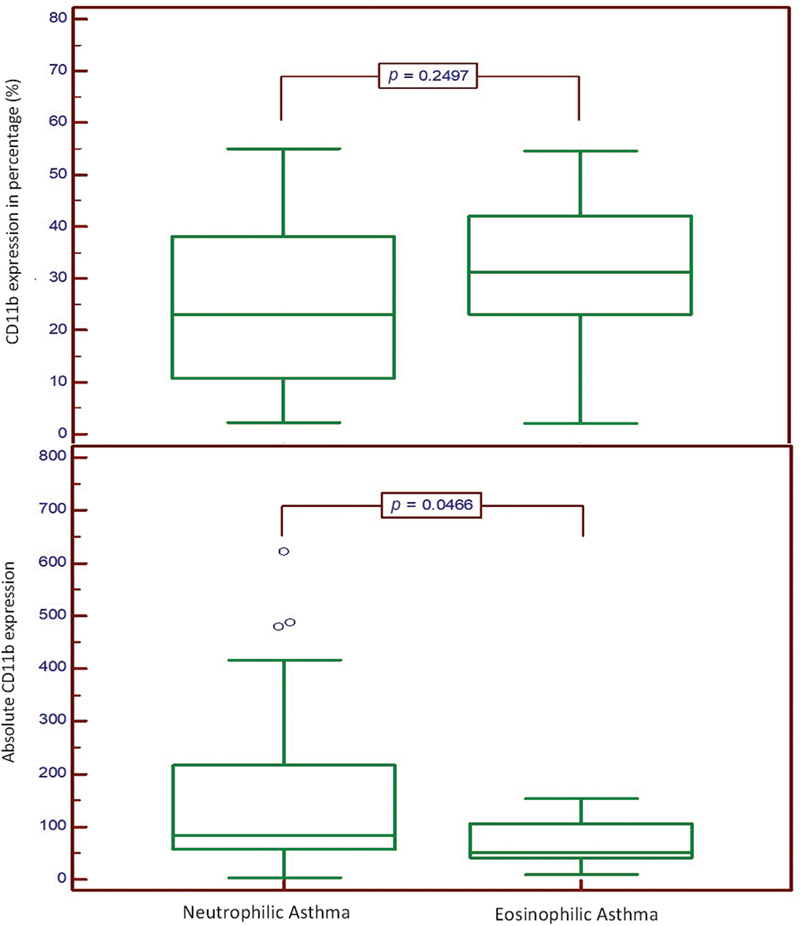
Comparison of CD11b expression in percentage and absolute number between subjects with neutrophilic asthma and eosinophilic asthma. Box plot comparison of CD11b expression in neutrophilic asthma and eosinophilic asthma. The upper box plot shows CD11b expression as a percentage, with no significant difference observed between the groups. The lower box plot shows absolute CD11b expression, with a significant difference identified between the groups.

The flow cytometry was done to measure the expression level of CD11b, which is mainly expressed by neutrophils, so the gating was adjusted to neutrophils ( Fig. 1 ). There was no significant difference of CD11b expression level in relative number (percentage) between the two groups ( *p* = 0.2497), but the CD11b expression level in absolute number showed significant difference ( *p* = 0.0466) ( Fig. 2 ).

In multivariate analysis, we determined the factor that influences the CD11b expression level. No association was found between CD11b expression levels with age, sex, and body mass index (BMI).

## Discussion

This study showed that 70% patients are in the neutrophilic asthma group. This study also discovered that individuals exhibiting the neutrophilic asthma phenotype tend to display increased infection parameters in laboratory tests, indicated by higher white blood cell counts and a greater percentage of neutrophils. This finding aligns with another study's conclusion that neutrophilic asthma is frequently associated with bacterial infections in the airways. Bacterial infections in the airway tract lead to the release of inflammatory mediators and trigger an acute response to infection. 9 The predominant species identified within the airway bacterial community were *Moraxella catarrhalis* , or a member of the *Haemophilus* or *Streptococcus* genera. The overall abundance of these organisms correlated significantly and positively with the concentration of IL-8 and the count of neutrophils in sputum. 23 Another study showed that nasal microbiotas dominated by *Moraxella* species were associated with increased exacerbation risk, whereas *Streptococcus* species-dominated assemblages increased the risk of rhinovirus infection. 24 Colonization by these species was linked to longer durations of asthma and lower post-bronchodilator lung function. 12 13 25 These patients often exhibit poor responses to conventional asthma therapies. As a result, the administration of antibiotics is considered essential for managing these individuals. 26

CD11b expression level in this study was higher in the neutrophilic asthma group. Another study with adult asthmatic neutrophil subjects showed that neutrophils expressing CD35 and CD11b were increased in patients with severe steroid-dependent asthma compared with mild and moderate-to-severe asthmatics, and nonasthmatic volunteers. 11 Study on pediatric subjects shows similar results. Neutrophils given bronchoalveolar lavage fluid from neutrophilic asthma subjects with severe symptoms experienced an increased proinflammatory phenotype characterized by increased CD62L, increased CCL2, increased CD11b, decreased CD11c, increased CD16, and increased CD66b expression. 13

An increase in CD11b expression indicates an increase in neutrophil activity. CD11b plays a role in leukocyte adhesion and movement during inflammation, and it functions as a C3bi complement receptor involved in complement uptake. 15 16 CD11b is mainly expressed by neutrophil. Increased expression level of CD11b could be found in active neutrophil, which later trigger the expression of various inflammatory mediator. 27 A study observed that the surface density of the activation marker CD11b, which increases during priming via degranulation, was elevated in blood neutrophils following tumor necrosis factor stimulation in long-term smokers. 28 In mice, alterations in lung innate immune cells during endotoxin exposure, influenza infection, and in two genetic models of chronic obstructive lung disease were distinguishable based on the upregulation of CD11b in alveolar macrophages. 27 Additionally, in vitro stimulation of neutrophils with N-formyl-methionyl-leucyl-phenylalanine or lipopolysaccharide significantly enhanced reactive oxygen species production, as well as levels of CD11b, IL-8, and matrix metalloproteinase-9. 29 Recruiting neutrophils to the lungs in asthma might represent a typical reaction to pulmonary inflammation.

The expression of CD11b is the only marker that differs between the two types of asthma, indicating that CD11b plays a significant role in determining asthma type. Single-cell ribonucleic acid transcriptomic analysis reveals an increase in the number and activity of regulons in CD11bDC cells. 30 Linear regression analysis shows that CD11b expression is not associated with other factors such as age, gender, or BMI. Another study on healthy subjects found that race influences oxidative burst reactivity but not CD11b expression. 31 The elevated expression of CD11b could provide insights into the mechanisms driving airway inflammation and remodeling. This marker has the potential to be explored as a therapeutic target for modulating immune activation, reducing asthma exacerbations, and improving long-term outcomes.

The study's relatively small sample size may limit the statistical power and reliability of the results. With a smaller group, it becomes more challenging to detect subtle effects or draw conclusions that can be confidently generalized to a larger population. Further study with more subjects is needed to validate the results. The absence of longitudinal data is a recognized limitation of our study. Without it, we are unable to assess trends or changes over time, which restricts our ability to draw causal inferences or understand the long-term implications of the phenomena studied. We strongly encourage future studies to adopt longitudinal designs to build upon our findings. While we made efforts to minimize the influence of confounding factors through careful study design and statistical controls, we acknowledge that not all relevant factors could be accounted for. This limitation may have influenced our results and interpretations. Future research could address this by incorporating more comprehensive approaches, such as advanced statistical models.

## Conclusion

Our study demonstrates that CD11b expression is elevated in the neutrophilic phenotype compared with the eosinophilic phenotype in pediatric asthma patients, suggesting its potential role in airway inflammation and remodeling. However, we recognize certain methodological limitations, including a limited sample size and the absence of longitudinal data, which may affect the generalizability of our findings. Future research should address these limitations to strengthen the evidence base.

CD11b shows promise as a biomarker for distinguishing asthma phenotypes, with potential clinical applications in personalized treatment strategies. Nonetheless, further studies are needed to validate its utility and assess its feasibility in clinical settings. Larger, longitudinal studies could clarify CD11b's role in the pathophysiology of asthma and its integration into diagnostic and therapeutic frameworks. By expanding on these findings, future research could pave the way for innovative therapies targeting immune modulation, reducing asthma exacerbations, and improving long-term outcomes for pediatric patients.
